# Megaesophagus

**DOI:** 10.11604/pamj.2021.38.138.27937

**Published:** 2021-02-08

**Authors:** Danilo Coco, Silvana Leanza

**Affiliations:** 1 Department of General Surgery, Ospedali Riuniti Marche Nord, Pesaro (PU), Italy,; 2 Department of General Surgery, Carlo Urbani Hospital, Jesi, Ancona, Italy

**Keywords:** mega esophagus, achalasia, computed tomography scan

## Image in medicine

Achalasia is a disorder of the primary motility of the esophagus characterized by insufficient relaxation of the lower part esophageal sphincter and absence of esophageal peristalsis, clinically it presents dysphagia for solids and liquids and “bird-beak” appearance on endoscopic and radiological studies. A 77-year-old man presented at our emergency room with a ten year history of dysphagia for solids and liquids, regurgitation and substernal chest pain after meals. He reported vomiting. Her medical history included arterial hypertension. She had no a previous surgical history of any type of surgery. In the past, he had refused any kind of treatment. He had a Glasow coma scale (GCS) of 15. His vital signs showed hypertension with arterial blood pressure of 180/100 mmHg, not tachycardia and not fever. Routine blood investigations not showed leukocytosis, normal hemoglobin and protein chain reaction (PCR) in the range. Arterial blood gas was normal. Upon physical examination, thoracic auscultation revealed decreased vesicular breath sounds in both hemithorax. Abdominal examination was unremarcable. A chest radiography revealed an air-fluid level. Thoracic-abdominal computed tomography (CT) scan demonstrated mega-esophagus with an air-fluid levels, a dilated and tortuous esophageal lumen and numerous food ingestis. Upper gastrointestinal endoscopy showed a dilated esophagus, food ingestis and mucosal ulcers. The patient refused operation but we made a multidisciplinary meeting to discuss how to treat other future cases like this.

**Figure 1:**
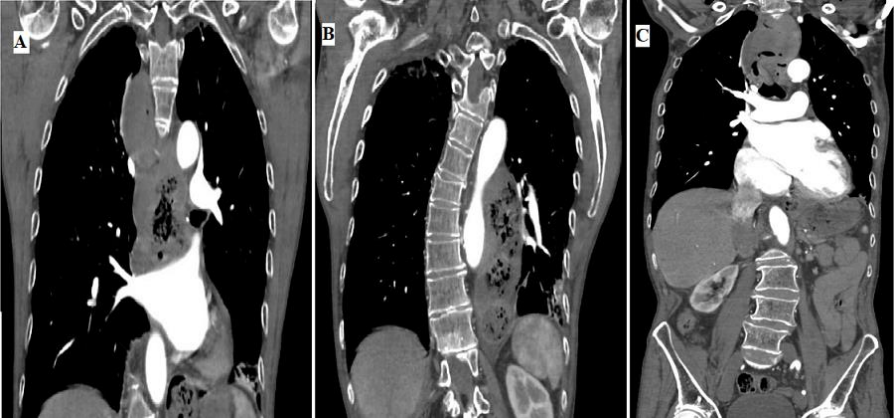
A) thoracic-abdominal computed tomography (CT) scan demonstrated mega-esophagus with an airfluid levels; B) thoracic-abdominal CT scan demonstrated a dilated and tortuous esophageal lumen; C) thoracic-abdominal CT scan demonstrated numerous food ingestis

